# Effect of Weight-Bearing and Mobilization After Treatment With Microfracture in Osteochondral Lesions of the Talus in Rabbits

**DOI:** 10.7759/cureus.70761

**Published:** 2024-10-03

**Authors:** Semih Ak, Bekir E Kilinc, Olcay Eren, Ahmet O Akpolat, Alev Cumbul, Baris Yilmaz

**Affiliations:** 1 Orthopaedics and Traumatology, Health Science University, Fatih Sultan Mehmet Training and Research Hospital, Istanbul, TUR; 2 Orthopaedic Surgery and Traumatology, Health Science University, Fatih Sultan Mehmet Training and Research Hospital, Istanbul, TUR; 3 Histopathology, Yeditepe University, Istanbul, TUR; 4 Orthopaedic Surgery, Health Science University, Fatih Sultan Mehmet Training and Research Hospital, Istanbul, TUR

**Keywords:** microfracture, mobilization, osteochondral lesion, talus, weight-bearing

## Abstract

Background: The aim of this study is to comparatively evaluate the effect of early weight bearing on recovery after treatment with the microfracture (MF) method in a rabbit model of osteochondral lesions (OCLs) of the talus.

Materials and methods: The study was carried out on 24 tali of 12 white rabbits. Experimental animals were divided into two groups. Rabbits whose weight-bearing and mobility were not limited after MF surgery were called Group 1, and rabbits with limited mobility were called Group 2. After 28 days of postoperative follow-up, tissue samples obtained from the tali were evaluated macroscopically according to the International Society for Cartilage Research Repair Evaluation scoring system 1 (ICRS-1) and microscopically according to the ICRS-2 scoring system.

Results: The mean ICRS-1 score was 87.5 ± 12.5 in Group 1 and 40.2 ± 7.8 in Group 2. The intergroup comparison of ICRS-1 scores showed that the ICRS-1 scores were significantly higher in Group 1 (p = 0.01). The mean ICRS-2 score was 74.3 ± 1.9 in Group 1 and 35.2 ± 1.9 in Group 2. The comparison of ICRS-2 scores between the groups showed that the ICRS-2 scores were significantly higher in group 1 (p = 0.01).

Conclusion: Early weight-bearing and mobility have a more favorable effect on cartilage healing after treatment of OCL of the talus with MF surgery.

## Introduction

Osteochondral lesion (OCL) of the talus is among the important causes of ankle pain [1]. When talus OCL is not treated appropriately, it can lead to problems such as chronic pain, osteoarthritis, and decreased range of motion [2]. Rest, plaster immobilization, use of nonsteroidal anti-inflammatory drugs (NSAIDs), physical therapy, and rehabilitation exercises are among the conservative treatment options. Excision, curettage, excision and curettage, antegrade or retrograde drilling, curettage and microfracture (MF), and curettage and autogenous grafting are surgical options that have been described for the treatment of OCL of the talus [3].

Arthroscopic or open MF surgery and the use of scaffolds after MF surgery have become popular surgical treatment methods in recent years [4]. During postoperative follow-up, many researchers immobilized the ankle joint with a splint for three weeks and did not allow weight-bearing [5]. Although good clinical results have been reported during follow-up, there are studies in the literature indicating that lesions in the load-bearing areas of the talus where cartilage thickness is high heal closer to normal. No clear evidence has been reported regarding the effect of immediate weight-bearing on recovery during the postsurgical follow-up period [6,7]. The putative positive effects of weight-bearing, especially on postoperative recovery, remain an issue that requires serious consideration.

In this study, we hypothesized that early mobility and weight-bearing contribute positively to recovery after MF surgery in the treatment of talus OCL.

The aim of this study was to comparatively evaluate the effect of early weight-bearing on recovery after MF surgery in a rabbit model of OCL of the talus.

## Materials and methods

An application requesting permission to conduct the present study was submitted to Yeditepe University Faculty of Medicine Experimental Research Center, and ethics committee approval was subsequently obtained (decision number: 2021/11-2). The study was conducted in terms of Based Declaration guidelines.

The animals were owned by the institution, and informed consent was obtained from the institution committee to use the animals in our study.

Similar studies on this subject were examined, and a sample size of n = 12 and a 90% confidence interval were used. The effect size of the study is sufficient with a value of 0.43. 

Experimental animals were divided into two groups. After the procedure, rabbits without restricted mobility and weight-bearing were included in Group 1, and the cast applied and range of motion restricted were included in Group 2.

Surgical procedure

**Figure 1:**
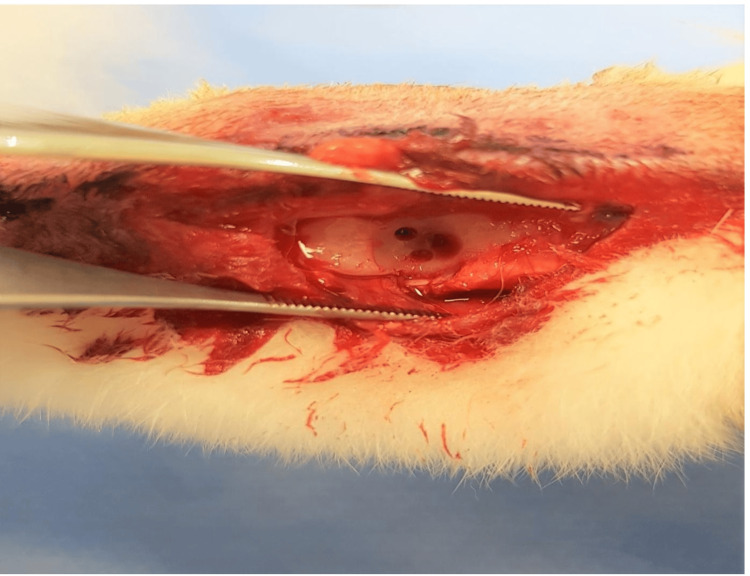
Microfracture was applied to the osteochondral lesion of the talus

The surgical procedure was performed according to the model described by Makitsubo M [8,9]. Experimental animals were anesthetized by intramuscular administration of 40 mg/kg body weight (BW) of ketamine and 10 mg/kg BW of xylazine. Cefazolin sodium (10 mg/kg BW) was administered prophylactically. The hair on both ankles was shaved off. The field was sterilized with a 10% povidone-iodine solution. The left ankle was entered with an anterior incision. The tibialis anterior tendon was retracted laterally, and the extensor retinaculum was opened to reach the talus. The surface of the talar cartilage was exposed by keeping the ankle in plantar flexion, and an OCL with a diameter of 3.0 mm and a depth of 4.0 mm was created. The MF was created at three points using a 0.7 mm Kirschner (K) wire with a 1 mm distance from each other (Figure 1).

**Figure 2:**
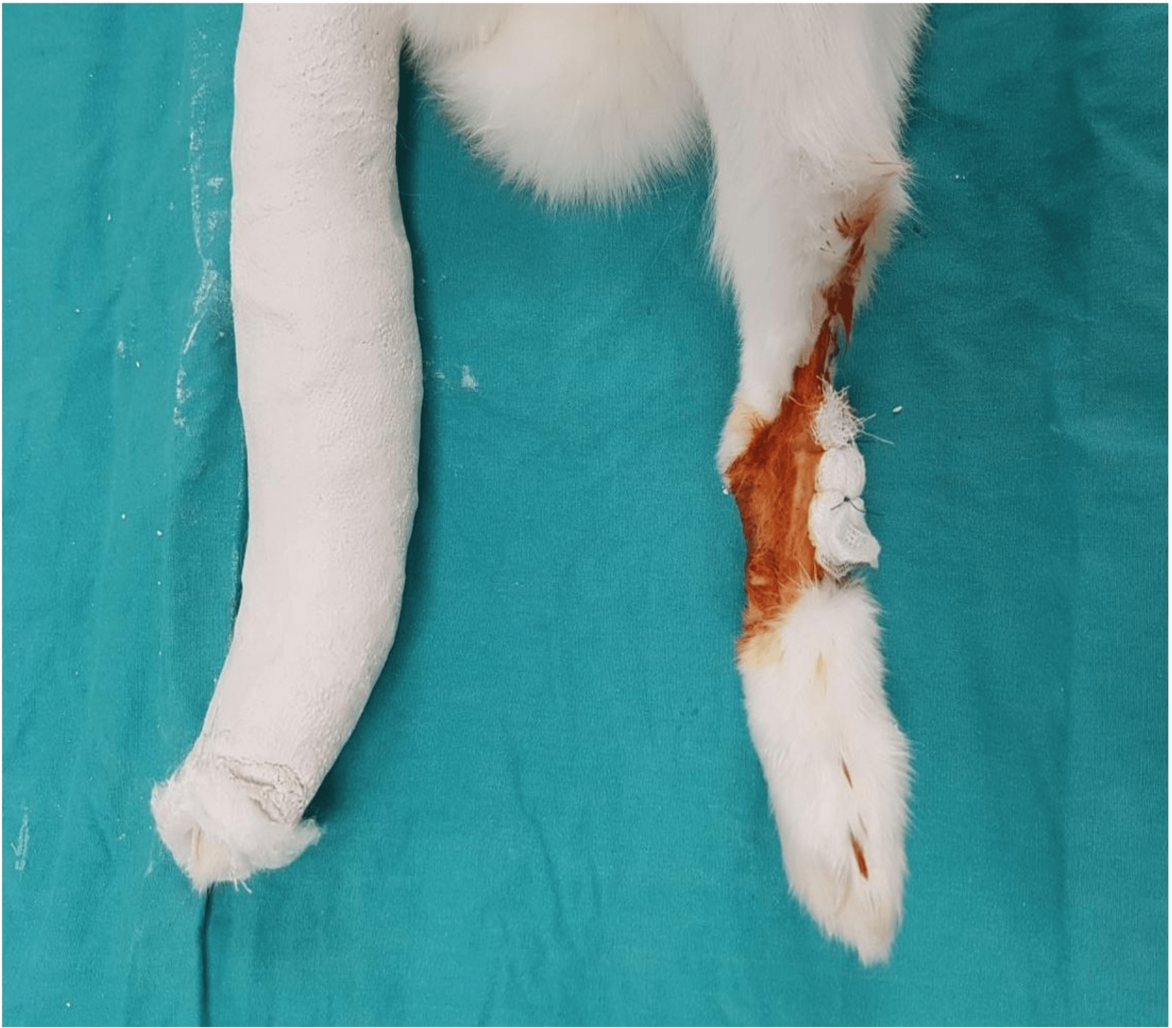
Weight-bearing was not allowed on one side of the extremity by applying a cast

During the closure of the surgical field, the extensor retinaculum was repaired, the skin was closed with prolene suture, and a sterile dressing was performed. After dressing, a circular cast was applied with the knee extended and the ankle in 30 degrees plantar flexion. In this way, the talus of the rabbit was prevented from carrying a load [10]. The other side was released to allow mobility and weight-bearing (Figure 2). For the first seven days after surgery, cefazolin sodium was continued at a dose of 10 mg/kg BW, and ketoprofen was administered at a dose of 3 mg/kg BW intramuscularly as an analgesic. At the end of the 28th day, the rabbits were sacrificed. The previous incisions were used to reach the tali. Using a mosaicplasty graft recipient, an osteochondral block (diameter, 6.0 mm; depth, 30 mm) containing the lesion in the center was removed [11].

Macroscopic examination

Tissue samples were examined by two different researchers, one of whom is an orthopedics and traumatology specialist, and the other is a histology and embryology specialist, in a double-blind manner, at different times, according to the International Society for Cartilage Research Repair Evaluation scoring system 1 (ICRS-1) scoring system.

Microscopic examination

**Figure 3:**
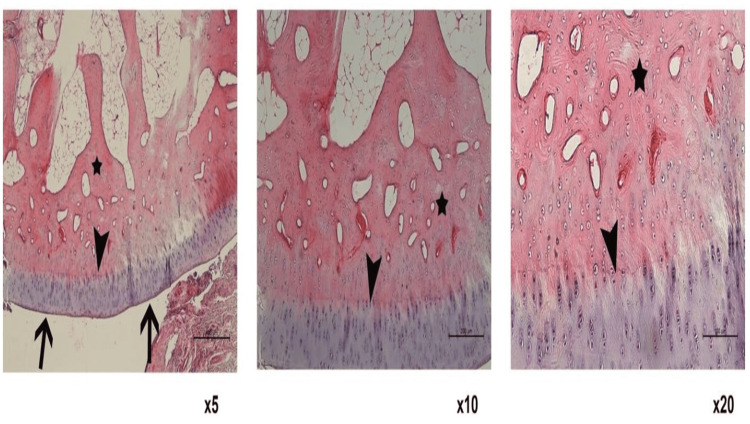
Histological image of Group 1. Black arrows (on 5X magnification) indicate the cartilage surface, black arrowhead indicates tidemark, and black arrow indicates bone areas. The smooth surface of the cartilage tissue and prominent tidemark are observed in the image.

**Figure 4:**
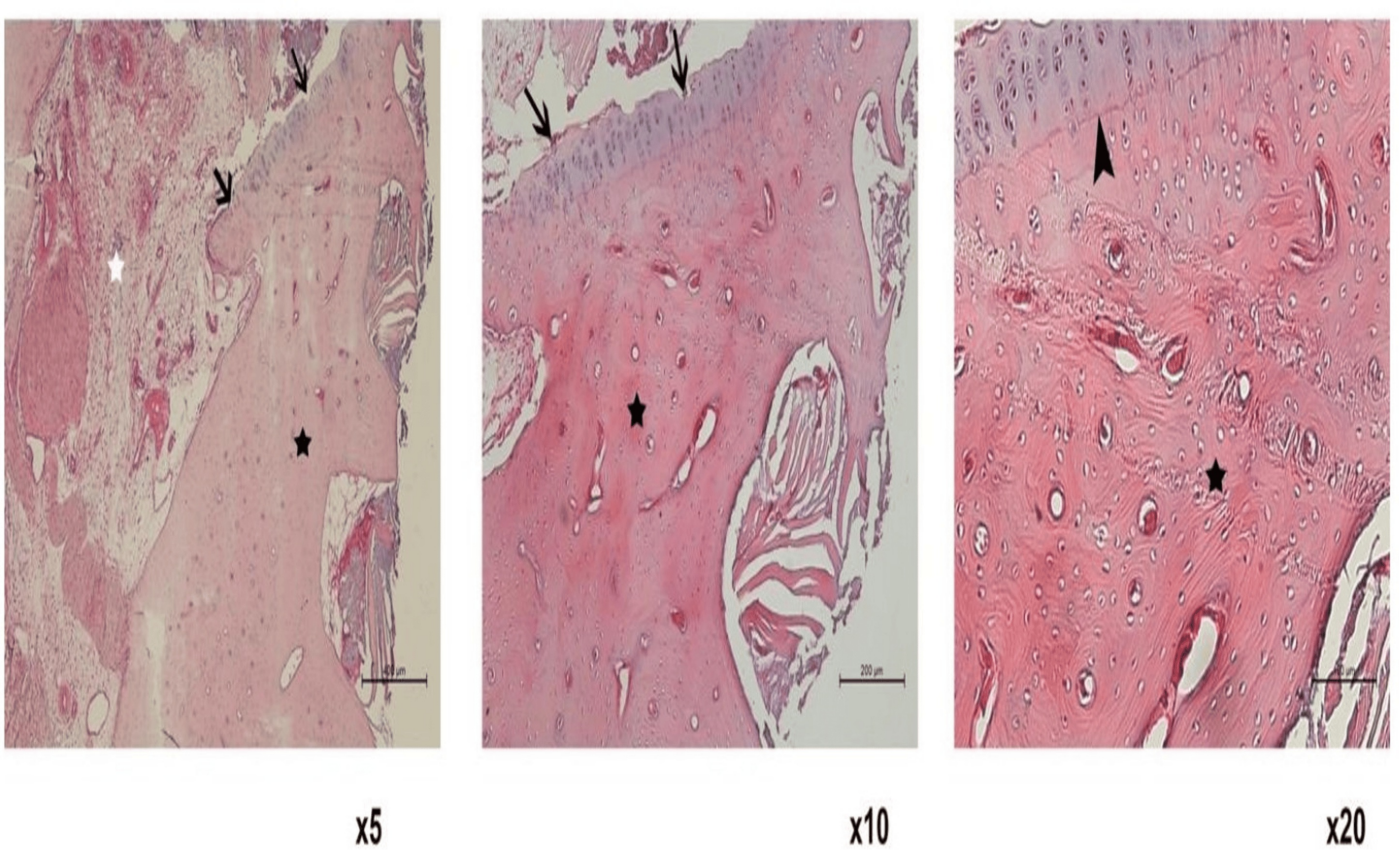
Histological image of Group 2. The black arrows show the cartilage surface, the black arrowhead indicates tidemark, the black star indicates the bone areas, and the white star indicates the inflammation in the synovial area.

Tissue samples were stained with the hematoxylin and eosin method after decalcification and examined and photographed under the Leica DM 6000 microscope with the help of the Leica Application Suite software. The scoring was performed according to the ICRS-2 classification (Figures 3, 4). The data obtained from the groups were compared statistically.

Statistical analysis

Descriptive statistics are presented as mean ± standard deviation for continuous variables. The conformity of the data to the normal distribution was evaluated with the Kolmogorov-Smirnov test. The homogeneity of variances between the groups was evaluated using Levene's test. Non-parametric tests were used in the study because the data did not show normal distribution (p = 0.01) and the number of data was below 30. The Wilcoxon test was used to compare Group 1 and Group 2. The ICRS-1 score was evaluated using the Wilcoxon test and intragroup correlation coefficient (ICC) to test the agreement between the observers. The relationship between measurements was evaluated using Spearman’s correlation coefficient. In the study, a p-value of <0.05 was considered statistically significant. The data were analyzed using the IBM SPSS Statistics for Windows, Version 25.0 (IBM Corp., Armonk, NY) on the computer.

## Results

Macroscopic examination

It was observed that the evaluations of the two investigators for the ICRS-1 score of Group 1 and Group 2 were similar (p = 0.32, p = 0.27, respectively). In addition, a high level of interobserver correlation was observed in the evaluation of the two groups (p = 0.32) (r ICC = 0.78, r ICC = 0.81).

The comparison of the ICRS-1 scores between the groups revealed that the ICRS-1 scores were significantly higher in Group 1 (p = 0.01) (Table 1).

**Table 1:** ICRS-1 and ICRS-2 scores of groups and comparison between the groups ICRS-1: International Society for Cartilage Research Repair Evaluation scoring system 1, ICRS-2: International Society for Cartilage Research Repair Evaluation scoring system 2

	Group 1 Mean ± SD	Group 2 Mean ± SD	p
ICRS-1 score	87.5 ± 12.5	40.2 ± 7.8	0.01*
ICRS-2 score	74.3 ± 1.9	35.2 ± 1.9	0.01*

Microscopic examination

A statistically significant difference was found between the ICRS-2 scores of Group 1 and Group 2. It was determined that the difference was due to the fact that Group 1 measurements (74.3 ± 1.9) were higher than Group 2 measurements (35.2 ± 1.3) (p = 0.01) (Table 1).

Tissue morphology, matrix staining, and chondrocyte aggregation scores were significantly higher in group 2, whereas surface architecture, basal integration, tidemark formation, vascularization, surface evaluation, deep layer evaluation, subchondral bone anomaly, inflammation and abnormal calcification, and general evaluation scores were significantly higher in Group 1 (p = 0.01 for all) (Table 2).

**Table 2:** ICRS-1 subscores in the groups and comparison between the groups ICRS-1: International Society for Cartilage Research Repair Evaluation scoring system 1

	Group 1 Mean ± SD	Group 2 Mean ± SD	p
Tissue morphology	0.83 ± 1.95	62.92 ± 7.82	0.01*
Matrix staining	9.58 ± 7.22	15.00 ± 5.22	0.01*
Chondrocyte aggregation	1.67 ± 3.39	90.42 ± 4.98	0.01*
Surface architecture	98.33 ± 3.89	49.58 ± 3.96	0.01*
Basal integration	87.5 ± 8.66	16.25 ± 3.77	0.01*
Tidemark formation	85 ± 35.03	5.42 ± 1.44	0.01*
Vascularization	85.2 ± 14.8	5.4 ± 7.2	0.01*
Surface evaluation	70.6 ± 24.6	20.8 ± 5.2	0.01*
Deep layer evaluation	90.2 ± 4.8	30.2 ± 7.4	0.01*
Subchondral bone anomaly	85.42 ± 8.38	17.5 ± 5	0.01*
Inflammation	91.25 ± 5.69	12.92 ± 3.34	0.01*
Abnormal calcification	92.5 ± 3.99	23.75 ± 6.78	0.01*
General evaluation	9.33 ± 1.42	7.83 ± 1.13	0.01*

For Group 1, there was a moderate negative correlation between tissue morphology, matrix staining, chondrocyte aggregation measurements, and ICRS-1 scores (p = 0.03). It was observed that there was a moderate positive correlation between surface architecture, tidemark formation, and inflammation measurements and the ICRS-1 score (p = 0.03). There was no significant correlation between abnormal calcification measurements and the ICRS-1 score (p > 0.05) (Table 3).

**Table 3:** Evaluation of the relationship between ICRS-1 and tissue morphology, matrix staining, chondrocyte aggregation, surface architecture, tidemark formation, inflammation, and abnormal calcification measurements ICRS-1: International Society for Cartilage Research Repair Evaluation scoring system 1

	Group1 ICRS-1 score		Group 2 ICRS-1 score	
	r	p	r	p
Tissue morphology	−0.52*	0.03	0.34	0.28
Matrix staining	0.54*	0.03	0.48*	0.04
Chondrocyte aggregation	−0.52*	0.03	−0.11	0.74
Surface architecture	0.52*	0.03	0.49*	0.04
Tidemark formation	0.49*	0.04	0.32	0.30
Inflammation	0.45*	0.04	−0.04	0.90
Abnormal calcification	0.28	0.38	−0.32	0.30

For Group 2, a moderate positive correlation was observed between the ICRS-1 scores and matrix staining and surface architecture measurements (p = 0.03). There was no significant correlation between tissue morphology, chondrocyte clustering, tidemark formation, inflammation, and abnormal calcification measurements and ICRS-1 scores (p > 0.05) (Table 3). 

## Discussion

This study observed the positive effects of early weight-bearing and mobility on cartilage healing at the macroscopic and microscopic levels after MF surgery in a rabbit model of OCL of the talus.

In recent years, with the developments in imaging methods, talus OCL has begun to be diagnosed more frequently [12]. The healing potential of cartilage tissue is quite limited due to its unique avascular and aneural structure. In the case of cartilage damage, chondrocytes proliferate and try to repair and regenerate the damaged area [13]. However, chondrocytes need nutrients to proliferate. To meet its needs, nutrients and other components must be delivered to the damaged area from the subchondral region. Therefore, injuries that do not reach the subchondral region are unlikely to heal spontaneously [13].

Although success rates in the treatment of osteochondral defects in the talus have increased with the emergence of different treatment methods in recent years, there are still issues open to research. There is no common opinion in the literature, especially regarding the rehabilitation process after MF [14-16]. In this study, we aimed to examine a controversial issue in the literature regarding early weight-bearing and early joint movement after rehabilitation with a randomized controlled animal experiment.

Among the bone marrow stimulation techniques, MF is a very effective method especially in the treatment of lesions less than 10 mm in diameter [16]. In some studies, it has been reported that MF treatment can be applied as a first-line surgical treatment in OCL of the talus, with satisfactory mid-term outcomes and improvement in clinical and functional scores [17-19]. In the long-term follow-up of patients who underwent MF alone after sustaining OCL of the talus, the reported success rate was 93% and 85.7% of the patients returned to sports [20]. However, factors such as the size and location of the lesion, the presence of a cyst or bone marrow edema, and obesity affect the success of treatment [17]. The present study used an animal model to reduce the risk factors that may affect recovery. In this way, we evaluated the effectiveness of our treatments in a more standardized fashion with the aim of reducing the factors that may affect the treatment.

In the literature, studies evaluating patients with talus OCL who were weight-bearing restricted for two to six weeks after MF surgery alone or MF surgery combined with adjuvant therapy have yielded different results [14,16,21,22]. Singh et al. reported a significant increase in ankle scores and a decrease in subchondral cyst sizes in patients who received plasma rich in growth factors after arthroscopic debridement and MF, but they stated that the procedure did not prevent the development of ankle osteoarthritis (21). However, Lee et al. reported that MF and MF+atelocollagen applications had similar long-term results in terms of pain, function, and histopathological scores (15). D'ambrosi et al. evaluated patients who received intra-articular adipose tissue-derived stem cell injection in addition to MF surgery and reported a significant improvement in clinical ankle scores [16]. Adjuvant therapy was not used in this study. However, more favorable histopathological results were observed with early weight-bearing and rapid range of motion in this study. This finding led us to question the necessity of additional treatment in the perioperative period. However, since the current study is an experimental animal study, it does not have the capacity to address this issue further.

There is only one study in the literature evaluating early weight-bearing after MF surgery for OCL of the talus. However, in this study, the ankles of the patients were fixed with a splint for at least two weeks and weight-bearing was not allowed. At the end of the study, the pain and functional scores of the patients who were allowed to bear weight were found to be better than those who were allowed to bear weight in the late period (four weeks) [23]. In this regard, when patients with ankle trauma were evaluated in terms of early weight-bearing, it was reported that early weight-bearing reduced the incidence of complications [24].

There are also some limitations in the present study. Although most patients with OCL of the talus have a chronic background, the model created in the study is an acute type of OCL. Histopathological planning of the study and working on experimental animals are other weaknesses. In addition, short-term outcomes were examined in our study, and long-term follow-up data were not included. The fact that the MF method used in the current model is the same as that used in chronic lesions, the anatomy of the rabbit talus being similar to the human talus, and rabbit physiology being similar to the human physiology makes the weaknesses in the present model negligible.

## Conclusions

Both macroscopical and microscopic scores were better with early weight-bearing. This finding is consistent with the literature data. In the treatment of talus OCLs with MF, the positive results of early weight-bearing and early movement on recovery will be beneficial in preventing the negative results due to immobilization, which is usually applied after surgery.
